# In Vitro Evaluation of Facial Pressure and Air Leak with a Newly Designed Cushion for Non-Invasive Ventilation Masks

**DOI:** 10.3390/healthcare8040523

**Published:** 2020-12-01

**Authors:** Hui-Ling Lin, Yu-Chi Lee, Ssu-Hui Wang, Li-Ying Chiang, Jui-Fang Liu

**Affiliations:** 1 Department of Respiratory Therapy, Chang Gung University, Taoyuan 33301, Taiwan; 2 Department of Respiratory Therapy, Chiayi Chang Gung Memorial Hospital, Chiayi 61363, Taiwan; ahui@cgmh.org.tw; 3 Department of Respiratory Care, Chang Gung University of Science and Technology, Chiayi 61363, Taiwan; chin9688@yahoo.com.tw; 4 Department of Industrial Design, School of Design, South China University of Technology, Guangzhou 510006, China; liyuqi@scut.edu.cn; 5 Department of Respiratory Therapy, Tungjen Hospital, Taipei 23141, Taiwan; amychiang0@gmail.com

**Keywords:** non-invasive ventilation, interface, cushion, facial pressure, air leaks

## Abstract

*Background:* The aim of this study was to evaluate the effect of a newly designed foam cushion on the air leakage and pressure when applied to the face. *Methods:* A teaching manikin connected to a bilevel positive airway pressure ventilator attached to four different brands of oronasal masks (Amara, Mirage, Forma, and Wizard) was used. The foam cushions of 5-mm and 10-mm-thickness were attached to the masks, and each mask was tested without a cushion. Six pressure sensors were placed on the manikin’s face, and data were recorded. Inspiratory volume and air leak flow from the ventilator were observed. *Results:* Air leakage was influenced by both the mask brand and the presence of a cushion. The presence of a cushion did not affect the Wizard mask in terms of leakage (*p* = 0.317) or inspiratory volume (*p* = 0.726). The Wizard and Amara masks generated the lowest contact pressure on the frontal forehead (*p* < 0.001) compared to the other five points. *Conclusions:* Utilisation of a cushion reduces air leakage and maintains greater inspiratory volume regardless of its thickness. The contact pressure varies depending on the brand of the mask, which would require a difference in the thickness of the cushion for pressure reduction.

## 1. Introduction

Non-invasive ventilation (NIV) is widely used in critical settings for treating patients with acute respiratory failure [1,2]. The utilisation of NIV reduces the length of intensive care unit stay as well as the intubation rate, further reducing medical expenses and mortality within five years [3]. NIV has been used to manage dyspnoea symptoms in patients who are terminally ill. Reports from Europe have illustrated that NIV is used to alleviate the symptoms of dyspnoea in approximately half of the patients with “do not resuscitate” status [4,5]. Additionally, long-term home NIV has been well established for chronic diseases in the last few decades [6].

NIV establishes a closed ventilation system with various types of patient interfaces, such as the nasal, total face, and oronasal masks, among which the latter are the most commonly used [7,8,9]. The type of interface has been reported as a key component in the success of NIV therapy. However, a review of studies comparing ventilator settings and outcomes reveals limited scientific focus on the selection and improvement of interfaces [6]. Inappropriate interfaces cause patient discomfort/pain, air leakage, and skin damage, resulting in the failure of NIV [8,10]. Studies have shown that approximately 10–38% of patients undergoing NIV refuse treatment because of discomfort caused by the interface and patient–ventilator asynchrony by the mask leak [9]. 

A nasogastric (NG) tube is often used to relieve gas distention in the stomach caused by NIV gas. However, the presence of an NG tube may further induce mask leak through the space between the patient’s face and mask; clinicians tend to tighten the mask, thus increasing pressure on the patient’s face. Consequently, patients undergoing NIV therapy often experience pressure ulcers, pain, discomfort, and anxiety. A previous study revealed that 2–23% of patients undergoing NIV therapy reported pain and skin breakdown caused by the mask after a couple of hours of therapy, and 70% of these patients reported severe pressure ulcers after two days [11].

Clinicians use different dressings, such as silicone foam, water/polyethylene oxide hydrogen, and cloth dressings, to reduce the skin breakdown caused by the NIV interface. Weng experimented on the pressure reduction achieved by different types of dressing and reported that the material of dressings significantly affects the timing of pressure ulcer formation [12]. Dellweg et al. studied pressure changes between the skin contact and air-filled masks and found that an increase in pressure from contact with the mask was correlated with an increase in inspiratory pressure [13]. Additionally, Maruccia et al. reported that elderly patients are at a high risk of pressure ulcers during NIV therapy; thus, dressings should be used alongside the NIV interface to reduce the risk of pressure ulcers and increase patient comfort [14]. However, most adhesive or foam dressings are packed in a square shape, which does not fit the patient’s face or the oval shaped NIV interface. Additionally, adhesive dressings are difficult to remove and can further induce skin breakdown during removal. Recently, Shikama et al. developed a customised NIV mask created with three-dimensional scanning technology to reduce the incidence of erythema, discomfort level, and contact pressure [15]. However, the devices are not readily available.

Therefore, we designed a cushion for oronasal masks with latex memory foam. The objective of this study was to evaluate the effect of cushion thickness on mask leakage and pressure applied to the patient’s face in a laboratory setting.

## 2. Materials and Methods

### 2.1. Cushion Material and Development

A cushion requires elasticity to absorb pressure and must be non-toxic to avoid skin irritation; therefore, we chose a non-formaldehyde laboratory-certified latex foam material that has antimicrobial properties against *Staphylococcus aureus* and *Escherichia coli* and is free from metallic and toxic substance residues. For easy application of the cushion and oronasal mask to a patient’s face, the foam was designed to be hollow and oval-shaped and wrapped in cotton cloth. To compare the effects of the cushion thicknesses, the following three conditions were tested: (1) no cushion, (2) 5-mm-thick cushion, and (3) 10-mm-thick cushion. 

### 2.2. Masks Choices

**Figure 1:**
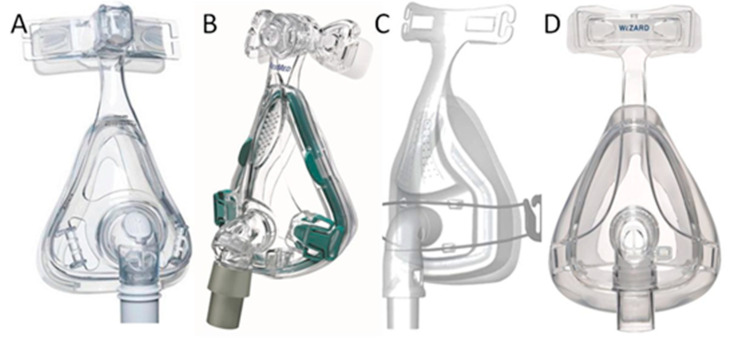
Appearance of the four masks: (**A**) Amara mask, (**B**) Mirage Quattro mask, (**C**) Forma mask, and (**D**) Wizard mask.

The four most commonly used oronasal masks in Taiwan were used: (A) Amara mask (Philips Respironics Corp., Murrysville, PA, USA); (B) Mirage Quattro mask (ResMed Corp., San Diego, CA, USA); (C) Forma mask (Fisher & Paykel Healthcare, Auckland, New Zealand); and (D) Wizard mask (Apex Medical Corp., Taipei, Taiwan), as shown in Figure 1. The mask sizes were chosen according to the manufacturer’s instructions.

### 2.3. Lung Model

**Figure 2:**
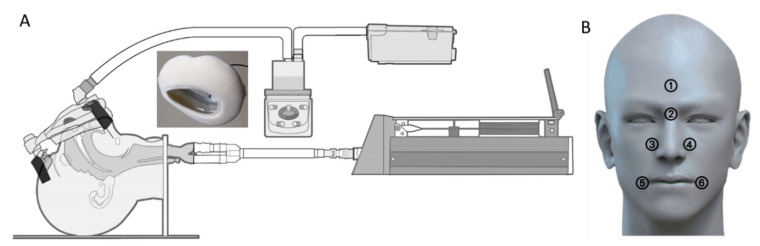
(**A**) Diagram of the experiment apparatus, (**B**) pressure measurement points.

A Philips Respironics bilevel positive airway pressure ventilator (BiPA Synchrony^®^, Philips Respironics Inc., Murrysville, PA, USA) was used to test the masks, with the following ventilator settings: spontaneous/timed mode; inspiratory positive airway pressure, 16 cm H_2_O; expiratory positive airway pressure, 6 cm H_2_O; breathing rate, 12 breaths/min. The ventilator measured and calculated parameters such as respiratory rate, tidal volume, minute volume, and leak flow rate. A single-compartment adult test lung (Michigan Instruments Inc., Grand Rapids, MI, USA) with a lung compliance of 100 mL/cm H_2_O and airway resistance of 5 cm H_2_O/L/s was connected. The mask was connected to a 180 cm single-limb ventilator circuit without a heated wire (VADI Medical Technology Co., Ltd., Taoyuan, Taiwan), and a dialled-heated humidifier (MR 410, Fisher & Paykel Healthcare, Auckland, New Zealand) was used at the highest temperature setting. Figure 2A shows the experimental apparatus.

### 2.4. Measurement of Pressure on the Face and Air Leak Volume

A mask was placed on a teaching manikin (Sakamoto Model Corp., Kyoto, Japan) by a well-trained respiratory therapist with three years of clinical experience who was blinded to the results. The tightness of the headgear was adjusted to allow the total leak rate (intentional and unintentional leak) <60 L/min in each condition, according to the manufacturer’s recommendations and Brill [16]. Two cushions with different thicknesses (5 mm and 10 mm) were attached to the chosen mask. Six pressure sensors (Hong-Zam Technology Co., Ltd., Taipei, Taiwan) were attached to the manikin at the forehead, nose bridge, nose edges, and corners of the mouth, as shown in Figure 2B. Pressure data from the six points were detected and recorded continuously for 30 min. In addition, inspiratory volume and air leak flow, measured by the ventilator, were observed and recorded every 5 min for 30 min. The experiments with three conditions and four masks were repeated three times in a random order. 

### 2.5. Statistical Analysis

Data was analysed using SPSS Version 23 (IBM Inc., New York, NY, USA). The data of each group are presented as mean ± standard deviation. The pressure at the six points, flow rate of air leak, and inspiratory volume were assessed using an analysis of variance, and differences between the groups were determined using Scheffee’s post hoc comparison. Significant differences were determined by a *p*-value < 0.05.

## 3. Results

### 3.1. Effect of a Cushion on Air Leak and Ventilation Volume

Table 1 shows the air leak and inspiratory volume of the four masks with and without the cushions. The results showed that air leakage was influenced by both the mask brand and the presence of a cushion. Applying a cushion of either 5 mm or 10 mm improves leak flow and inspiratory volume in the Amara, Mirage, and Forma masks, and the Amara mask with a 5-mm cushion created less air leakage and higher inspiratory volume (*p* < 0.05) than other masks. The presence of the cushion did not affect the Wizard mask in terms of air leakage (*p* = 0.317) or inspiratory volume (*p* = 0.726).

**Figure 3:**
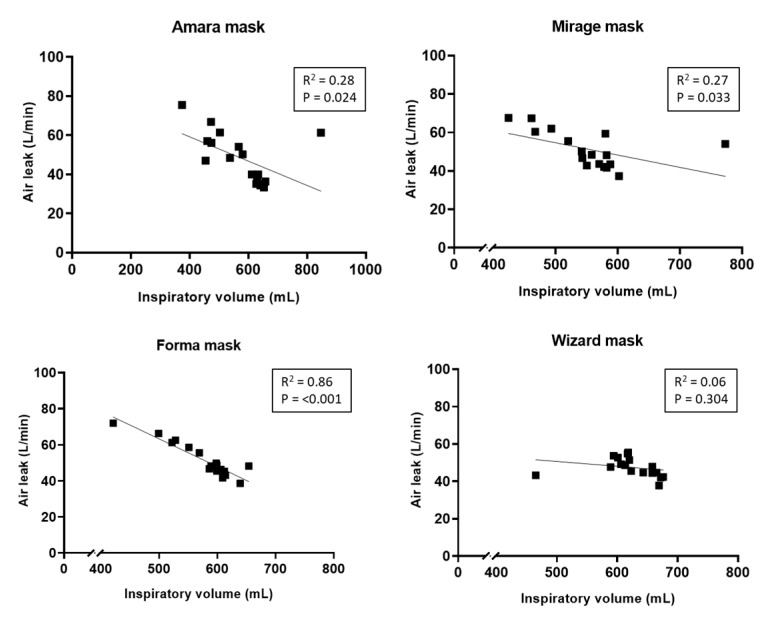
Correlations between air leak and inspiratory volume among the four masks.

Air leakage from the Wizard mask was significantly lower than the other three masks (*p* = 0.001), but the overall inspiratory volume was similar among the groups (*p* = 0.057). The 5-mm cushion significantly improved air leakage from the Forma mask (*p* = 0.002); however, no significant difference in inspiratory volume was observed (*p* = 0.061). Figure 3 illustrates the correlation between air leakage and inspiratory volume among the four masks. Pearson correlation showed a significant moderate negative linear relationship between leak flow and inspiratory volume (r^2^ = −0.607; *p* < 0.001).

### 3.2. Impact of a Cushion to the Pressure on the Face

**Figure 4:**
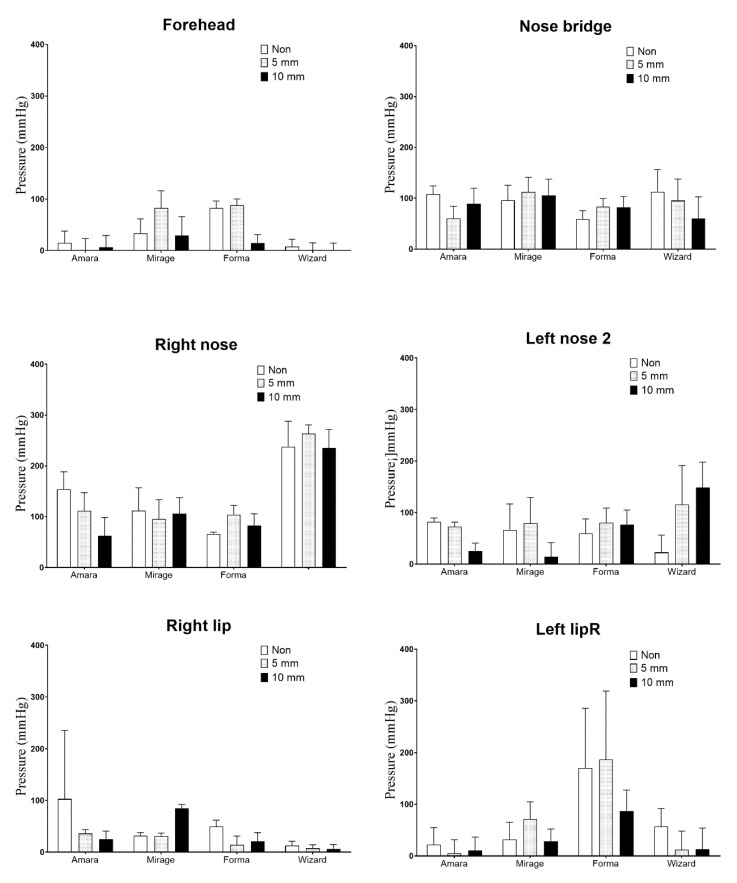
Pressure on each detection point from the four masks with and without a cushion.

Figure 4 shows a comparison of the pressure applied to the face at the detection points. The Wizard mask generated a significantly lower contact pressure on the forehead and right hip, but significantly greater pressure on the right nose than the other three masks. The pressure on the face caused by the mask with and without the cushion varied. The Wizard and Amara masks generated the lowest pressure on the frontal forehead (*p* < 0.001) compared to the other five points. The pressure on the bridge and left side of the nose was approximately 100 mm Hg. The pressure applied by the Wizard mask to the right side of the nose was significantly higher than that of the other three masks (*p* < 0.001). Overall, the Wizard mask generated the lowest pressure on the face, and the Forma mask generated the highest pressure, but the 10-mm cushion significantly reduced the pressure (*p* < 0.001).

**Figure 5:**
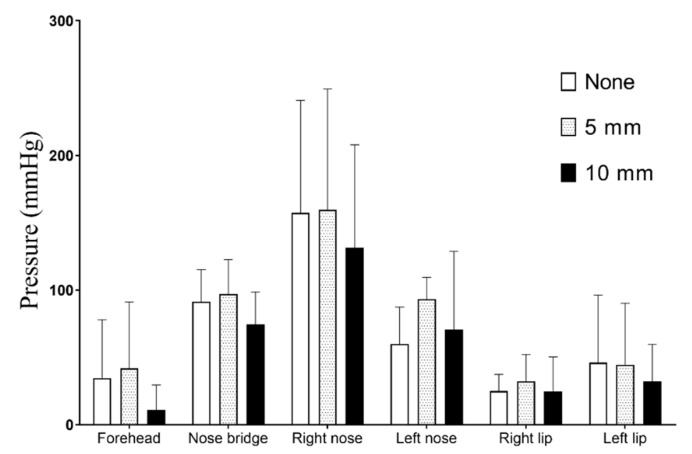
Average pressure applied to the face at each point.

Figure 5 illustrates the average pressure applied to all masks with and without cushions. The pressure on the right side of the nose was three-fold higher than that at the frontal forehead and corners of the mouth. The pressure at the frontal forehead (*p* = 0.041), bridge of the nose (*p* = 0.033), and left corner of the mouth (*p* = 0.048) was significantly reduced by using the 10-mm cushion. The Wizard mask resulted in the highest pressure both with and without the cushion, and the pressure on the right side of the nose was approximately 237.5 ± 49.9 mm·Hg. The pressure on the face was influenced by both the brand of the mask and thickness of the cushion. For the Amara mask, the 10-mm cushion achieved the optimal decompression effect. Conversely, the pressure from the Mirage and Wizard masks increased with both the 5-mm and 10-mm cushions.

## 4. Discussion

The results of the study illustrate that utilisation of a cushion reduces air leakage and maintains greater inspiratory volume. The contact pressure varies depending on the brand of the mask, which would require a difference in the thickness of the cushion for pressure reduction.

### 4.1. Influence of the Cushion on Ventilation

The presence of leaks, particularly large unintentional leaks, may affect the triggering and cycle-off phases, resulting in patient-ventilator asynchrony [17]. Leakage during expiration can lead to loss of positive end-expiratory pressure, and an increase in ventilator auto-triggering. The inspiratory leaks can prolong the inspiratory time and the lead to reduced inspiratory sensitivity; therefore, greater inspiratory efforts are required. While most NIV ventilators are capable of compensating for air leakage within a certain range, substantial leaks will result in increased inspiratory flow for leak compensation to maintain the set pressure, which may cause side effects, including impairing the mask seal and dry air gas. 

Under the same tightness conditions, the Wizard mask without a cushion generated significantly lower air leakage and higher inspiratory volume than the other three masks. The Amara, Mirage, and Forma masks with the 5-mm cushion had significantly lower leakage, and the inspiratory volume increased by nearly 100 mL. Masks designed for patients of different ethnicities are speculated to influence the fitting of the mask to the face. Our teaching manikin was designed in Japan with Asian facial features, such as a smaller/flatter face, a smaller nose, and wider cheekbones. The Amara, Mirage, and Forma masks were designed in America, Australia, and New Zealand, respectively, for a better fit for Caucasian patients, and the Wizard mask was designed in Taiwan for Asian patients, designed with a smaller top and narrower bottom. A larger air leak from both sides of the nose and corners of the mouth was observed with the other three masks. However, the use of a cushion reduced the amount of air leak and increased inspiratory volume. Thus, masks should be loosened to reduce the pressure applied to the patient’s face while maintaining an acceptable amount of air leakage. A preliminary review of the literature revealed no studies on NIV interfaces for Asian faces. Further clinical studies are required to confirm this. 

### 4.2. Pressure Caused by NIV Masks 

A previous study reported that the incidence of pressure ulcers associated with medical equipment in an intensive care unit was approximately 2–6%, which was 20–47% of the pressure ulcers in the entire hospital [18]. The primary cause of pressure ulcers is excessive pressure applied to thinner areas of subcutaneous tissue, leading to blockage of perfusion under the area. For example, pressure ulcers over the tibia area are formed when the pressure exceeds 40 mm Hg [19]. Clinically, pressure ulcers caused by NIV masks mostly appear around the bridge of the nose. Previous randomised clinical trials revealed that the pressure generated by an NIV mask on the bridge of the nose reached 322–532 mm Hg [20,21,22]. Our study revealed a pressure as high as 263 mm Hg, which indicates that our simulation study is relevant. 

Human factors might contribute to the difference in pressure. Pressure on both sides of the nose and both corners of the mouth can differ considerably, possibly as a result of improper fitting when the operator adjusts the mask. Because this study was performed by a well-trained respiratory therapist, data were collected only after confirming the proper fit of the mask; therefore, this study effectively controlled for human error. However, our data revealed asymmetrical pressure, which may have resulted from the greater force applied by the dominant hand of the operator when adjusting the mask headgear. A pressure sensor could be used during the training of proper mask fitting to achieve even force while tightening the oronasal mask. 

Brill et al. reported that different brands of NIV masks exert significantly different pressures on the bridge of the nose [20]. Our data also illustrated the different pressures exerted on the face by NIV masks. Overall, the Amara exerted the lowest pressure, followed by the Mirage and Forma masks, with the Wizard mask exerting the highest pressure. The shape of the mask affected the pressure at each detection point. The pressure may have been affected by the characteristics of each product. The Wizard mask features a thicker plastic (or silicone) material to enable deformation and facilitate a better fit to different facial shapes. However, excessively thick plastic is prone to creating wrinkles and creases, which may exert force unevenly. 

A previous study demonstrated that NIV settings and the contact area between the face and mask affect the pressure exerted on the skin [13]. Our study revealed that the thicker, 10-mm cushion was the most effective at reducing the pressure exerted on the face. However, the design must be further improved to enhance the benefits of the cushion when used with the Mirage and Wizard masks, such as combining foam and silicone. A redesigned mask may enhance user comfort and reduce the pressure exerted on the face.

This study had a few limitations. First, data obtained from a single manikin cannot represent the pressure exerted by a mask on the faces of all patients. Patients receiving NIV in Taiwan have experienced unintentional leakages and skin breakdown from an inappropriately fitted mask. Therefore, this particular Asian-feature manikin was chosen to mimic the intended study population. However, everyone is born with different facial structures. Hence, studies on different facial features are recommended. Furthermore, only two cushion thicknesses were chosen. During the pilot study, four thicknesses of foam pads (3, 5, 10, and 15 mm) were tested. The 3-mm cushion absorbed humidification from the gas, and the foam collapsed once it was wet within a short period of time. The 15-mm cushion was too thick to fit the contour of the face and masks, causing greater air leak than that without the cushion. Therefore, the study used 5 and 10-mm cushions for the final evaluation. Additional thicknesses of cushions with different materials are warranted. Finally, the human facial structure is composed of different amounts of soft tissue, which would be a layer of cushion against the NIV mask. The contact pressure in our bench testing with a manikin may be overestimated. However, the results provide clinicians with a reference for patterns of pressure exerted on different points of the face by NIV masks.

## 5. Conclusions

The shape of the Asian manikin results in a greater amount of air leakages when using a mask designed for Caucasians, which can be reduced by combining a cushion, thereby improving ventilation. However, the contact pressure on the face caused by NIV masks can be reduced by applying a cushion. The required thickness of the cushion varies depending on the original design of the oronasal mask. In addition, the headgear may cause uneven pressure distribution, which is possibly due to the dominant hand use of the operator. The results of the present study suggest that when fitting a mask focus should be on ensuring symmetrical pressure and tightness. Clinical trials with patient feedback regarding the cushion are warranted.

## Figures and Tables

**Table 1:** Leak flow and inspiratory volume among the four masks with and without a cushion.

Mask/Condition	Leak Flow (L/min)	Inspiratory Volume (mL)
**Amara**		
None	61.3 ± 8.9 *	557.3 ± 160.1
5-mm cushion	35.8 ± 2.3	643.3 ± 10.9
10-mm cushion	47.3 ± 8.6	527.3 ± 77.4
*p*-value	0.205	0.493
**Mirage**		
None	62.0 ± 4.7 *	490.8 ± 54.3
5-mm cushion	43.9 ± 4.6	574.3 ± 23.3
10-mm cushion	47.5 ± 4.7	593.7 ± 89.1
*p*-value	0.005	0.247
**Forma**		
None	62.7 ± 5.8 *	515.0 ± 52.0
5-mm cushion	45.5 ± 5.6	618.0 ± 23.1
10-mm cushion	45.2 ± 2.39	599.7 ± 10.9
*p*-value	0.004	0.039
**Wizard**		
None	47.4 ± 4.1	633.8 ± 26.6
5-mm cushion	42.9 ± 5.8	604.3 ± 75.2
10-mm cushion	49.4 ± 4.9	628.5 ± 32.2
*p*-value	0.317	0.726

* Significantly greater than the other two factors.
